# Adaptive constraints at the range edge of a widespread and expanding invasive plant

**DOI:** 10.1093/aobpla/plad070

**Published:** 2023-11-05

**Authors:** Rebecca A Fletcher, Daniel Z Atwater, David C Haak, Muthukumar V Bagavathiannan, Antonio DiTommaso, Erik Lehnhoff, Andrew H Paterson, Susan Auckland, Prabhu Govindasamy, Cornelia Lemke, Edward Morris, Lisa Rainville, Jacob N Barney

**Affiliations:** School of Plant and Environmental Sciences, Virginia Tech, 1015 Life Science Circle, Blacksburg, VA 24061, USA; Department of Animal & Range Sciences, Montana State University, 103 Animal Biosciences Building, Bozeman, MT 59717, USA; School of Plant and Environmental Sciences, Virginia Tech, 1015 Life Science Circle, Blacksburg, VA 24061, USA; Department of Soil and Crop Sciences, Texas A&M University, 370 Olsen Boulevard, College Station, TX 77843, USA; School of Integrative Plant Science, Section of Soil and Crop Sciences, Cornell University, Ithaca, NY 14853, USA; Department of Entomology, Plant Pathology, and Weed Science, New Mexico State University, MSC 3BE, Las Cruces, NM 88003, USA; Plant Genome Mapping Laboratory, University of Georgia, 111 Riverbend Road, Athens, GA 30602, USA; Plant Genome Mapping Laboratory, University of Georgia, 111 Riverbend Road, Athens, GA 30602, USA; Department of Soil and Crop Sciences, Texas A&M University, 370 Olsen Boulevard, College Station, TX 77843, USA; Division of Agronomy, ICAR-Indian Agricultural Research Institute, New Delhi 110012, India; Plant Genome Mapping Laboratory, University of Georgia, 111 Riverbend Road, Athens, GA 30602, USA; Department of Entomology, Plant Pathology, and Weed Science, New Mexico State University, MSC 3BE, Las Cruces, NM 88003, USA; Plant Genome Mapping Laboratory, University of Georgia, 111 Riverbend Road, Athens, GA 30602, USA

**Keywords:** Biotic interactions, fitness trade-off, flowering time, invasive species, range edge, range limits, *Sorghum halepense*

## Abstract

Identifying the factors that facilitate and limit invasive species’ range expansion has both practical and theoretical importance, especially at the range edges. Here, we used reciprocal common garden experiments spanning the North/South and East/West range that include the North American core, intermediate and range edges of the globally invasive plant, Johnsongrass (*Sorghum halepense*) to investigate the interplay of climate, biotic interactions (i.e. competition) and patterns of adaptation. Our results suggest that the rapid range expansion of Johnsongrass into diverse environments across wide geographies occurred largely without local adaptation, but that further range expansion may be restricted by a fitness trade-off that limits population growth at the range edge. Interestingly, plant competition strongly dampened Johnsongrass growth but did not change the rank order performance of populations within a garden, though this varied among gardens (climates). Our findings highlight the importance of including the range edge when studying the range dynamics of invasive species, especially as we try to understand how invasive species will respond to accelerating global changes.

## Teaser

Invasive species often face novel abiotic and biotic interactions at their range edge that may limit further expansion into new geographies or elicit fitness trade-offs that do not occur in the range core. Using one of the world’s worst weeds, Johnsongrass, that is found across the Southern US grown in a large experiment across five US states, we found that expansion into new diverse environments was likely not the result of local adaptation. Contrary to the weedy nature of Johnsongrass, competition in the first year strongly limits performance of this invasive plant, especially at the range edge where populations are currently rare. Our study highlights the importance of understanding the factors limiting geographic expansion, in particular at the range edge, especially as we try to understand how invasive species will respond to climate change.

## Introduction

The factors that prevent species from continual range expansion have been of great interest to biologists for well over a century. In the absence of obvious physical barriers (e.g. a mountain range) that limit range expansion, a general framework has been proposed in which a combination of biotic, abiotic and demographic factors interact to set range limits (Angert 2009; Sexton *et al.* 2009; Stanton-Geddes *et al.* 2012; Csergő *et al.* 2017). Thus, populations persist along an environmental gradient until population growth is no longer sustainable (Hargreaves *et al.* 2014).

The ‘center-periphery hypothesis’ (Pironon *et al.* 2017) and the related ‘abundant center hypothesis’ (Sagarin and Gaines 2002) maintain that species are most abundant in the ‘core’ or centre of their range, and get progressively less common/abundant toward the range edge. This has served as the basis of numerous studies on diverse taxa. Range expansion is driven and limited by a combination of dispersal, population growth rate and density-dependent biotic factors (Thomas 2010), the interactions of which can be quite different at the range core versus at the range edge (Geber 2008). Populations at the range edge can be geographically distant from the range core and are often rare and occur at low densities (Gaston 1990). Thus, local adaptation is hindered by low connectivity and insufficient genetic variation (Colautti *et al.* 2010; Körner *et al.* 2016). Gene flow from core and intermediate populations (i.e. populations located between the core and edge) can increase genetic diversity in populations at the edge, but it can also have the detrimental effect of introducing maladaptive genes, hindering adaptation to conditions at the edge of the range (Kirkpatrick and Barton 1997; Holt *et al.* 2005; Bontrager and Angert 2019). Life history trade-offs can further constrain adaptation to conditions at the range edge (Holt *et al.* 2005; Sexton *et al.* 2009; Alexander and Edwards 2010; Chuang and Peterson 2016). However, the supporting evidence for these biogeographic hypotheses remains equivocal (Sagarin and Gaines 2002; Pironon *et al.* 2017).

Invasive species introduced to new geographies often undergo rapid range expansion, even as they face novel genetic, biotic and abiotic constraints on fitness (Theoharides and Dukes 2007; Alexander and Edwards 2010), and can serve as important systems to test elements of range limits and local adaptation (Sexton *et al.* 2009). For example, Colautti and Barrett (2013) found that, while the evolution of earlier flowering time in the invasive plant *Lythrum salicaria* enabled it to expand its range poleward, there was a trade-off of vegetative growth. However, even in invasive study systems, the relative contributions of abiotic (e.g. climate) and biotic (e.g. competition) factors on range limits are not well understood (Sexton *et al.* 2009).

At large spatial scales, climate is thought to be the main driver of species’ ranges, as demonstrated by decades of correlative and experimental studies (as reviewed in Sexton *et al.* 2009). At smaller scales, biotic effects such as competition, predation and pollination interact in setting geographic limits (Bullock *et al.* 2000; Sexton *et al.* 2009; Watling and Orrock 2010; Louthan *et al.* 2015). Biotic effects are often impacted by abiotic conditions, appearing to be most important in abiotically less stressful environments (Sanford *et al.* 2003; Louthan *et al.* 2017). Thus, biotic interactions may interact in complicated ways with climate to influence species population growth rates.

Here, we studied a widespread and range-expanding invasive perennial grass to identify factors that govern its continent-scale range expansion across two major climate axes (i.e. temperature North-to-South and precipitation East-to-West) that included the range edge. We asked the following questions: (i) Do climate and biotic factors interact to limit range expansion?; (ii) Is there evidence of adaptation to conditions along temperature and precipitation gradients? and (iii) Is there evidence that adaptation is constrained at the range edge? Given our previous experience with local populations (Atwater *et al.* 2018), we expected competition to strongly limit Johnsongrass performance, which we hypothesized would be strongest under less favourable growing conditions (i.e. range edges). We also expected ‘home’ Johnsongrass populations to generally outperform ‘away’ populations. Alternatively, if local adaptation is weak, this supports a previously demonstrated ‘general-purpose-genotype’ common in Johnsongrass populations (Atwater *et al.* 2015, 2018; Lakoba and Barney 2020), allowing strong performance across a range of growing conditions.

## Materials and Methods

### Study system

Johnsongrass [*Sorghum halepense*], thought to be native to western Asia (Paterson *et al.* 2020), is one of the most damaging agricultural weeds globally, commonly invading agricultural fields, roadsides, rights-of-way and natural ecosystems (Warwick and Black 1983; Rout *et al.* 2013). A single plant is capable of producing tens of thousands of seeds in a single growing season without any clear dispersal mechanism (likely gravity dispersed), and an extensive rhizome network (McWhorter 1961). Both seeds and rhizomes contribute to the invasiveness of Johnsongrass, as seeds are vital for spread (Atwater *et al.* 2017) and rhizomes allow local persistence and dominance once established (McWhorter 1961) with long-distance dispersal likely a result of contamination (e.g. mowers, harvesters). Further, pollen-mediated gene flow can contribute to the dispersal of adaptive alleles to adjacent Johnsongrass populations, leading to improved species persistence (Maity *et al.* 2022).

The core of Johnsongrass’s North American range occurs across much of the southeastern USA, with the original site of introduction in South Carolina likely in the mid-1800s, quickly spreading across the southern USA (Sezen *et al.* 2016). Currently, Johnsongrass population abundance declines sharply around 38°N and 102°W, which we define here as the ‘intermediate range’ because these populations likely experience different conditions and population dynamics compared with the range core and range edge (Chuang and Peterson 2016). Johnsongrass populations beyond the intermediate range are uncommon, small and isolated (DiTommaso and Lehnhoff personal observations), which we define as the range edge (Jump and Woodward 2003; Bridle and Vines 2007; Chuang and Peterson 2016). There is anecdotal evidence that Johnsongrass is unable to overwinter as rhizomes in the northern edge, and may be limited by arid conditions in the western edge (Warwick *et al.* 1984, 1986).

Recent studies show that Johnsongrass populations have diverged both phenotypically and genetically. Traits of Johnsongrass populations depend strongly on home climate and habitat, suggesting that local adaptation to varying environmental and climatic conditions along with phenotypic plasticity have played an important role in the success of this widespread invasive species (Atwater *et al.* 2015, 2018; Sezen *et al.* 2016; Lakoba and Barney 2020). For example, we have shown that Johnsongrass sampled from a small geographic region appears to rely on local adaptation in non-agricultural habitats and plasticity in agricultural systems (Atwater *et al.* 2018), but we have not tested this at large spatial scales across climate gradients. However, it remains unclear what role local adaptation plays in limiting Johnsongrass range expansion at large spatial scales, which is projected to move poleward with climate change (McDonald *et al.* 2009; Lakoba *et al.* 2021*a*). To elucidate the role that local adaptation plays in response to abiotic and biotic factors, we established five common gardens spanning the western and northern ranges of Johnsongrass using reciprocal transplants grown with and without local competition.

### Seed collections

We collected Johnsongrass seeds spanning the western and northern range of Johnsongrass near the garden sites. Across the North/South range we collected seeds from core (Georgia), intermediate (Virginia) and edge (New York) populations. Across the East/West range, we collected seeds from core (Georgia, same as above), intermediate (Texas) and edge (New Mexico) populations. Seeds from GA, VA and TX were collected between June and August 2011 and were subsequently planted in a greenhouse on the Virginia Tech campus in Blacksburg, VA where they were maintained as live germplasm. Cuttings from the plants were transplanted into a seed increase garden in May 2016, and mature seeds were harvested in August–October 2016. Seeds from the NY and NM populations were collected from the original source populations in August 2016 and were used directly in the present study. In all cases, seeds used in this experiment represent multiple maternal lines from a single source location. Using a mix of seed sources is not ideal, but we were limited logistically to when we received seeds for the edge populations. Results should be interpreted considering this variation.

In addition to the five populations, we included a control population (‘phytometer’) that is not from any of the garden locations and serves as a consistent ‘away’ population in all gardens. We are using this population as a phytometer *sensu*Strobl *et al.* (2018), ‘phytometers are indicator transplants that provide information on site conditions based on plant survival, growth and reproduction’. Seeds for the phytometer were purchased through a commercial vendor (Azlin Seed Service, 112 Lilac Dr., Leland, MS 38756), which were locally sourced in the Mississippi Delta area. We have used this phytometer in previous studies (Smith *et al.* 2021).

### Common gardens

We used a series of common gardens to investigate the interacting effects of abiotic (e.g. local climate), biotic (e.g. competition) and genetic (e.g. invader population source) factors on local adaptation, population growth and range limits. While we are not testing climate gradients per se, our gardens span large temperature (latitudinal) and precipitation (longitudinal) gradients, and we have consistently shown that Johnsongrass home climate strongly impacts performance (Atwater *et al.* 2015, 2018; Lakoba and Barney 2020). While three gardens along each axis are not sufficient to capture all variation across these large gradients, significant logistical challenges constrain the number of field sites and our study has a similar approach to Colautti and Barrett (2013) (and others) who established three gardens across a large climate gradient representing the existing core-to-edge range of an invasive plant.

Reciprocal transplant experiments are one of the most effective methods for investigating patterns associated with range dynamics (Blanquart *et al.* 2013), and by studying various life history traits, such as growth and flowering time, we can assess potential trade-offs that may lead to adaptive constraint. We used seedling transplants to standardize plant size and to test the survival and performance of the seedling stage.

We established five gardens near the locations of the five source populations (GA, VA, TX, NY and NM; Table 1) in areas where Johnsongrass populations are known to occur, but in garden plots that lacked the species. The common gardens were established in either March or May 2017, depending on the last frost date of the garden location (Table 1). We considered this to be a better test of site climatic effects than planting all gardens at the same time and thereby shortening the growing season at warmer sites. To improve germination, seeds from all populations were pre-treated by soaking them in 100 % bleach for 4 h followed by a 1-h rinse in tap water (Atwater *et al.* 2018). After pre-treatment, seeds were germinated in 128-well flats filled with Miracle-Gro^®^ Potting Mix. Seedlings were allowed to grow for approximately 5 weeks in a greenhouse located in Blacksburg, VA before being transplanted into each garden.

**Table 1.** Coordinates, elevation, mean annual temperature (°C; MAT) and total annual precipitation (mm; TAP) of the Johnsongrass source populations and common garden locations, including that for the 2017 experimental season. The last and first frost dates, experimental planting date and harvesting date are also shown for each of the five common gardens.

	Georgia			Virginia			Texas			New York			New Mexico		
	Population	Garden	2017	Population	Garden	2017	Population	Garden	2017	Population	Garden	2017	Population	Garden	2017
Latitude (DD)	33.883	33.729		37.194	37.194		31.060	30.552		42.764	42.451		32.202	32.314	
Longitude (DD)	−83.154	−83.302		−80.574	−80.302		−97.342	−96.428		−75.552	−76.461		−106.732	−106.745	
Elevation (m)	238	147		518	504		195	66		334	287		1177	1176	
MAT (°C)	16.2	16.5	18.1	11.2	11.6	12.1	19	20.1	21.7	6.6	7.5	8.8	16.2	17.5	18.5
TAP (mm)	1230	1220	1420	957	1013	1041	858	994	1326	1049	941	1080	234	213	294
Last Frost Date		24-Mar			4-May			2-Mar			14-May			1-May	
Planting Date		29-Mar			29-May			22-Mar			24-May			17-May	
First Frost Date		7-Nov			6-Oct			29-Nov			3-Oct			20-Oct	
Harvest Date		6-Nov			17-Oct			7-Nov			10-Oct			4-Oct	

Prior to the start of the experiment, each garden was tilled so that all transplants were planted into uniform, competition-free, bare ground. Each garden consisted of 10 replicate blocks with each block split into two plots. Each 8 × 4m plot within a block was randomly assigned to one of two treatments: a competition treatment or a bare-ground competition-free control treatment. The competition treatment was designed to evaluate one of the important biotic limitations to Johnsongrass establishment and performance; acknowledging that herbivory and pathogens are other important biotic interactions, but beyond the scope of this study. In the competition treatment, the resident flora was allowed to colonize naturally with no other management applied, and in all cases resulted in a dense weed flora as would be expected in an agricultural field. We have used this approach in similar studies where we are not interested in measuring competition per se (Atwater *et al.* 2017, 2018); rather we are mimicking what Johnsongrass would experience at these locations naturally as a seedling—strong competition with fast-growing resident weeds. The bare-ground treatment was maintained by manually removing weeds on a weekly basis. In each plot, one seedling from each of the five Johnsongrass populations and the phytometer were transplanted into a grid, randomly arranged with 2 m spacing between seedlings. Individuals from all populations were transplanted in all treatments, in all blocks and in all locations.

Transplants were given 2 weeks to establish in the field, during which we supplied supplemental water as needed to mitigate transplant shock. Seedlings that failed to establish were not included in the analyses (Georgia = 0, New Mexico = 21, New York = 14, Virginia = 5 and Texas = 0). After the establishment period, no additional water was added, except in New Mexico, because populations in New Mexico are almost always found in irrigated agricultural fields, irrigation and drainage ditches and other locations that have a consistent water supply during the growing season (Lehnhoff personal observation), we supplied the 10 experimental blocks in the New Mexico garden with minimal supplemental water (irrigation) on an as-needed basis through the duration of the experiment. In addition to the 10 blocks that received irrigation, we included five additional blocks that did not receive any supplemental water beyond the initial 2-week establishment period. Because of the limited sample size, the data collected from the five extra blocks in New Mexico were not assessed in the main analyses described below. More information and results from the no-irrigation treatment in New Mexico can be found in Appendix 3 of the Supplementary Materials. As an aside, since this experiment concluded we have observed Johnsongrass growing in the dry uplands in neighbouring Arizona (Barney personal observation) suggesting that Johnsongrass is capable of persistence in unirrigated portions of the study area.

Beginning 3 weeks after the establishment of each garden, we recorded the date of the first flower on a weekly basis for each Johnsongrass individual. We also began harvesting fully matured panicles to avoid seeds shattering onto the soil. Panicles were labelled and stored and included in the final aboveground biomass. Panicle phenotype varies a lot among Johnsongrass populations, and estimating or counting seeds was not feasible. More so, this is a perennial plant with both sexual and vegetative reproduction, so individual plant fitness is not simply a measure of seed production. Fortunately, aboveground biomass is a strong predictor of fitness (as reviewed in Younginger *et al.* 2017), and thus we will use biomass and height as proxies for performance. At the end of the growing season, as determined by the first frost date of each garden location (Table 1), we recorded survival and culm height. Aboveground biomass was harvested, dried at 60 °C for 3 days, and weighed. The following spring (2018), we recorded overwinter survival.

The weather of each garden in the experimental year of 2017 was slightly warmer and wetter than the long-term averages (Table 1) but trended the same across all gardens. While executed as a single large experiment, we considered our North–South and East–West gardens independently for several reasons. First, most climate gradient studies consider only a single axis, often temperature North–South, and analysing them separately is in line with this—that is, two separate gradients. Second, different mechanisms may be at play in response to temperature and precipitation that would potentially muddle interpretation if they were combined and analysed as core, intermediate and edge. Considering them separately allows for independent analyses and interpretations.

### Statistical analysis

Because we were interested in performance and flowering time along two different gradients, we analysed the gardens along the latitudinal (Georgia, Virginia, New York) and longitudinal (Georgia, Texas, New Mexico) gradients separately (note that data from the Georgia garden were included in the analyses of both gradients as the range core). In addition to recorded performance traits, we also calculated both aboveground biomass and height of each population relative to the phytometer within each garden by taking the logarithm of the ratio between the biomass or height value of each plant and the phytometer in each respective block and treatment (see Appendix 1 in the Supplementary Materials for more details). This allowed us to test for the performance of each Johnsongrass population to a consistent phytometer benchmark, which was always an ‘away’ population. We used mixed-effects linear models to analyse aboveground biomass (cube-root transformed to meet model assumptions), height, flowering time (Julian days from transplant day to first flower), natural-log transformed biomass relative to phytometer and natural-log transformed height relative to phytometer. All models were fit using the R package ‘lme4’ (Bates *et al.* 2015). To investigate our first two questions, (i) whether there is evidence that climatic and biotic factors interact to limit Johnsongrass range expansion and (ii) whether there is evidence of adaptation to conditions along range gradients, we included the fixed effects (predictor variables) of population, treatment, garden and their interactions. We also included the random effect of block. The effect of competition on Johnsongrass biomass was also calculated and analysed as above (see Supplementary Appendix 4). Significance was assessed at ∝ =0.05, and when a significant effect was present, we implemented pairwise contrasts using Tukey correction to compare levels within the predictor variable, as implemented in the package ‘emmeans’ (Lenth 2019). For significant interactions, we conducted pairwise comparisons within the levels of the interacting predictors. To investigate our third question, whether there is evidence of a trade-off between life histories that might constrain adaptation at the range edge, we assessed the relationship between height and flowering time by performing a linear regression with flowering time as a fixed effect and block as a random effect. All analyses were carried out in R Core Team (2018).

## Results

For clarity, the Johnsongrass populations will be labelled with state abbreviations (GA, VA, NY, TX, NM) while gardens will be labelled with full state names (Georgia, Virginia, New York, Texas, New Mexico). The phytometer is labelled as PHYT.

### Latitudinal gradient

**Figure 1.**
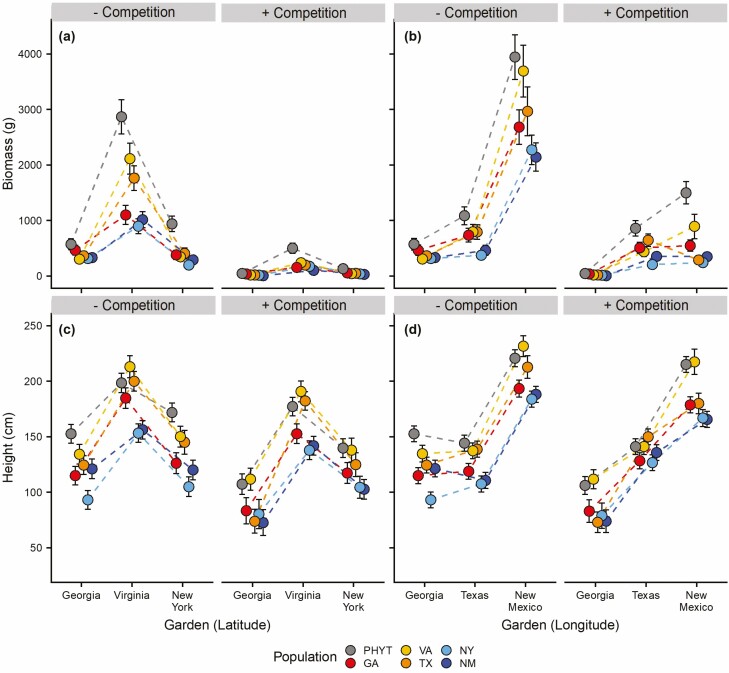
Effects of population, treatment and garden on biomass (back-transformed) along a latitudinal (a) and a longitudinal (b) gradient and height along a latitudinal (c) and longitudinal (d) gradient of Johnsongrass’s North American range. Separate mixed-effects models were performed for each of the two gradients. Points are estimated marginal means and error bars show standard error. See Table 2 for statistical details.

Without competition, we found that the PHYT produced the most biomass and grew the tallest, while the two edge populations (NY and NM) produced the least biomass and grew the shortest (Table 2; Supplementary Table S1; Fig. 1A and C) across all three gardens. We also found biomass and height of all populations were greatest in the Virginia (intermediate) garden compared to the Georgia (core) and New York (edge) gardens, where Johnsongrass performed about equally (Fig. 1A and C). There was a significant interaction between population and garden for biomass (Table 2). While the GA, TX and VA populations generally had similar biomass in New York and Georgia, the VA population had the greatest biomass in its home garden with the NY and NM edge populations performing the worst (Supplementary Table S1; Fig. 1A). Competition universally decreased both Johnsongrass height and biomass, but the effect of competition on both variables varied across gardens (Table 2; Fig. 1A and C). Competition greatly reduced biomass but did not change the rank order of Johnsongrass population performance (Fig. 1A). The impact of competition on Johnsongrass plant height was more modest relative to that of biomass, and likewise had relatively little change into the rank order (Fig. 1C). The results of both biomass and height relative to the PHYT followed similar patterns to those resulting from comparisons of the populations to the PHYT in the Tukey Tests (Supplementary Table S6; Fig. S4A and C). Detailed results of biomass and height relative to the PHYT are presented in Appendix 4 in the Supplementary Materials.

**Table 2.** Results of mixed-effects models testing the effects of population, garden, treatment and their interactions on cube-root biomass, height and flowering time along latitudinal and longitudinal gradients. All models included the random effect of block. The *F*-statistic was calculated using the Type III sum of squares. Values in bold are significant at *P* < 0.05.

	Latitudinal Gradient				Longitudinal Gradient			
	*SS*	*df*	*F*	*P*	*SS*	*df*	*F*	*P*
Cube-root Biomass								
Population	237.29	5	25.17	**<0.001**	273.65	5	23.82	**<0.001**
Garden	234.40	2	62.17	**<0.001**	770.80	2	167.71	**<0.001**
Treatment	1438.42	1	763.02	**<0.001**	1066.55	1	464.13	**<0.001**
Pop. × Gard.	56.16	10	2.98	**0.001**	71.24	10	3.10	**0.001**
Pop. × Treat.	18.39	5	1.95	0.087	8.04	5	0.70	0.624
Gard. × Treat.	50.69	2	13.44	**<0.001**	357.03	2	77.68	**<0.001**
Pop. × Gard. × Treat.	13.26	10	0.70	0.721	25.44	10	1.11	0.357
ln(Biomass Relative to Phytometer)								
Population	11.56	4	3.64	**0.007**	20.52	4	8.02	**<0.001**
Garden	1.51	2	0.95	0.401	0.45	2	0.35	0.705
Treatment	8.95	1	11.26	**0.001**	19.49	1	30.48	**<0.001**
Pop. × Gard.	8.36	8	1.31	0.239	8.83	8	1.73	0.094
Pop. × Treat.	3.18	4	1.00	0.409	4.12	4	1.61	0.173
Gard. × Treat.	0.98	2	0.61	0.542	7.55	2	5.90	**0.003**
Pop. × Gard. × Treat.	3.89	8	0.61	0.768	6.77	8	1.32	0.234
Height								
Population	86667	5	27.56	**<0.001**	59649	5	23.76	**<0.001**
Garden	108226	2	86.05	**<0.001**	308701	2	307.38	**<0.001**
Treatment	38796	1	61.70	**<0.001**	14393	1	28.66	**<0.001**
Pop. × Gard.	10470	10	1.66	0.090	10060	10	2.00	**0.034**
Pop. × Treat.	4129	5	1.31	0.259	2732	5	1.09	0.367
Gard. × Treat.	4606	2	3.66	**0.027**	28210	2	28.09	**<0.001**
Pop. × Gard. × Treat.	3785	10	0.60	0.812	5910	10	1.18	0.307
ln(Height Relative to Phytometer)								
Population	4.04	4	14.45	**<0.001**	2.43	4	10.93	**<0.001**
Garden	0.38	2	2.75	0.082	0.33	2	2.97	0.067
Treatment	0.04	1	0.58	0.448	0.05	1	0.84	0.362
Pop. × Gard.	0.18	8	0.33	0.954	0.52	8	1.16	0.322
Pop. × Treat.	0.15	4	0.54	0.708	0.10	4	0.44	0.777
Gard. × Treat.	0.01	2	0.04	0.963	0.47	2	4.24	**0.016**
Pop. × Gard. × Treat.	0.28	8	0.50	0.852	0.41	8	0.93	0.496
Flowering Time								
Population	3882	5	10.09	**<0.001**	3860	5	13.48	**<0.001**
Garden	111460	2	724.21	**<0.001**	202108	2	1765.08	**<0.001**
Treatment	98	1	1.27	0.260	243	1	4.24	**0.040**
Pop. × Gard.	792	10	1.03	0.419	1573	10	2.75	**0.003**
Pop. × Treat.	234	5	0.61	0.694	250	5	0.87	0.499
Gard. × Treat.	109	2	0.71	0.493	211	2	1.84	0.161
Pop. × Gard. × Treat.	317	10	0.41	0.940	285	10	0.50	0.890

**Figure 2.**
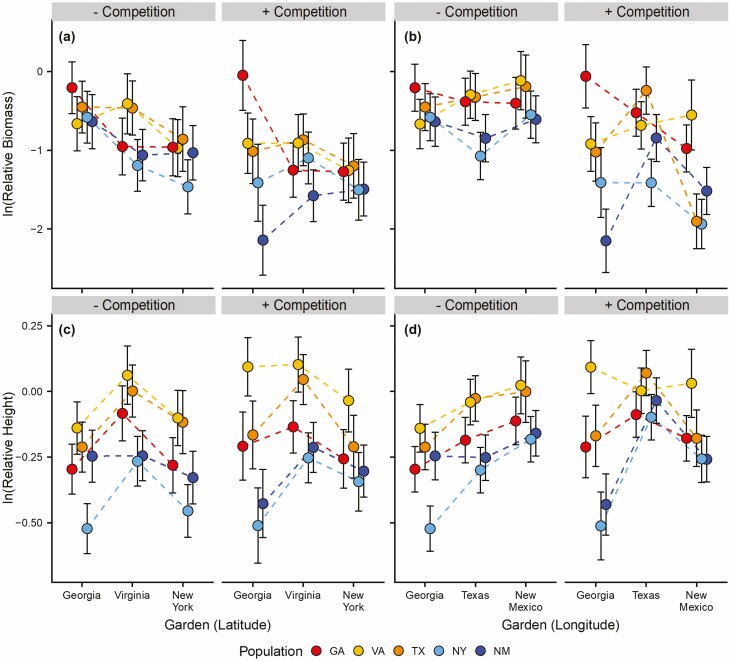
Effects of population, treatment and garden on ln(biomass relative to phytometer) along a latitudinal (a) and a longitudinal gradient (b), and ln(height relative to phytometer) along a latitudinal (c) and longitudinal gradient (d) of Johnsongrass’s North American range. Separate mixed-effects models were performed for each of the two gradients. Points are estimated marginal means and error bars show standard error. See Table 2 for statistical details.

Johnsongrass ln(biomass relative to PHYT) and ln(height relative to PHYT) varied among populations (Table 2; Fig. 2A and C). The ln(biomass relative to PHYT) was greatest for the GA population (Table 2) and smallest for the NM population (Table 1). On the other hand, the VA population had the greatest ln(height relative to PHYT) and the NY population had the smallest (Table 2). There was also a significant effect of weed competition on ln(biomass relative to PHYT) (Table 2), with ln(biomass relative to PHYT) being lower in the weed-competition treatment than the bare-ground treatment (Fig. 2A). We found no significant interaction between population and garden. The lnRR differed among gardens (Supplementary Table S6), and indicated competition from weeds was most intense in Georgia (lnRR ± SE = −3.39 ± 0.21) compared to Virginia (lnRR ± SE = −2.07 ± 0.17) and New York (lnRR ± SE = −1.99 ± 0.19) gardens (Supplementary Fig. S4); however, lnRR did not appear to vary among populations (Supplementary Table S6).

**Figure 3.**
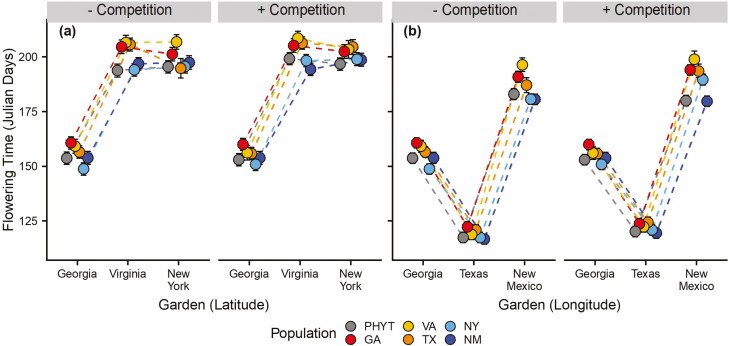
Effects of population, treatment and garden on Johnsongrass flowering time along a latitudinal (a) and a longitudinal gradient (b) of Johnsongrass’s North American range. Separate mixed-effects models were performed for each of the two gradients. Points are estimated marginal means and error bars show standard error. See Table 2 for statistical details.

**Figure 4.**
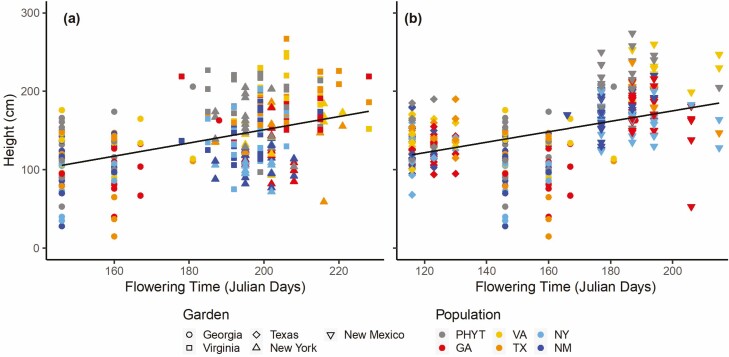
Results of linear regressions of the relationship between Johnsongrass height and flowering time along a latitudinal (a) and a longitudinal gradient (b) of Johnsongrass’s North American range. Separate mixed-effects regression models were performed for each of the two gradients. Points are estimated raw values and the black line is the model-predicted regression line.

We found significant variation in flowering time among gardens (Table 2) with plants in Georgia flowering on average 45 days earlier than plants in Virginia and New York (Fig. 3A). We also found a significant effect of population on flowering time (Table 2): along with the PHYT, the two edge populations (NY and NM) flowered 7 days earlier than that of GA and the two intermediate populations (VA and TX) populations (Fig. 3A; Supplementary Table S1). Results from the linear regression showed a positive relationship between height and flowering time (Fig. 4A; *P* < 0.001, *Estimate* = 0.84, *t* = 6.0, *df* = 80), suggesting a trade-off in that earlier flowering plants are shorter than later flowering plants; a change in nearly 100 % between early and late flowering individuals.

Plants in competition in Georgia suffered the highest mortality by the end of the growing season, with only 64 % of individuals surviving until the end of the season. Survival at the end of the season in the other two gardens was overall higher (Virginia: bare-ground = 93 %, weeds = 98 %; New York: bare-ground = 93 %, weeds = 88 %). Of the plants that survived to the end of the first growing season in Georgia, 74 % of those in bare ground and 62 % in weed competition survived the winter to re-emerge in the spring. There was 100 % rhizome winter kill in New York across all populations and treatments. In Virginia, of the plants that survived to the end of the season, 89 % of plants in bare ground and 72 % in weed competition survived the winter to re-emerge in the spring (see Supplementary Table S3 in Appendix 2 in Supplementary Materials for more details).

### Longitudinal gradient

The PHYT again accumulated more biomass than all other populations across all gardens, while the edge NY and NM populations performed worst. Competition decreased both biomass and height, but the magnitude of these effects varied among gardens (Table 2; Fig. 1B and D). Johnsongrass biomass was most strongly suppressed by weeds in New Mexico (Fig. 1B). In contrast, Johnsongrass height was less affected by competition, but did vary among gardens (Fig 1D). The results of both biomass and height relative to the phytometer followed similar patterns as those found in comparisons of populations to the phytometer in the Tukey Tests. Detailed results of biomass and height relative to the phytometer are presented in Appendix 4 in the Supplementary Materials.

Both ln(biomass relative to PHYT) and ln(height relative to PHYT) varied among populations (Table 2). The two edge populations had lower ln(biomass relative to PHYT) and ln(height relative to PHYT) compared to the other populations, GA and VA had the largest ln(biomass relative to PHYT), and VA had the largest ln(height relative to PHYT) (Fig. 2B and D; Table 2). Biomass and height relative to the PHYT also varied between treatments and among gardens (Table 2). We did not find evidence that ln(biomass relative to PHYT) was different between the weed-competition and bare-ground treatments in Texas, while in the other two gardens, weed-competition decreased ln(biomass relative to PHYT) (Fig. 2B and D; Table 2). On the other hand, ln(height relative to PHYT) appeared to be similar in both treatments in the Georgia and New Mexico gardens, but ln(height relative to PHYT) was greater in the bare-ground treatment than the weed-competition treatment in Texas (Fig. 2B and D; Table 2). Competition from weeds was most intense in the Georgia garden (lnRR ± SE = -3.40 ± 0.20) and least intense in the Texas garden (lnRR ± SE = -0.43 ± 0.14) (Supplementary Fig. S5).

Flowering time varied among populations and depended strongly on garden location (Table 2, Fig. 3). Johnsongrass flowered earliest in Texas, by 40 days compared to core Georgia and 80 days compared to edge New Mexico, but there were no differences in flowering time among populations within the Texas garden (Fig. 3B; Supplementary Table S2). Generally, in Georgia and New Mexico, the edge populations (NM, NY), along with the PHYT, flowered earliest, whereas VA and GA flowered the latest (Fig. 3B; Supplementary Table S2). There was also a significant effect of treatment on flowering time with plants in the bare-ground treatment flowering earlier than in the competition treatment (Table 2; Fig. 3B). As with the latitudinal gradient, the results from the linear regression of flowering time and height along the longitudinal gradient indicated a positive relationship (*P* < 0.001, *Estimate* = 0.80, *t* = 5.9, *df* = 79), suggesting a consistent association between shorter plants and earlier flowering (Fig. 4B).

Survival to the end of the season was high in both New Mexico (bare-ground = 100 %, weed-competition = 98 %) and Texas (100 % in both treatments). All plants that survived to the end of the growing season in both New Mexico and Texas survived the winter to re-emerge in the spring (see Supplementary Table S3 in Appendix 2 in the Supplementary Materials for more details).

## Discussion

We used five common gardens across large North/South and East/West environmental gradients spanning the core to edge range of Johnsongrass in the USA to explore the interactive effects of abiotic, biotic and adaptive factors on the range limits of a widespread invasive plant. Overall, our results suggest that Johnsongrass’s capacity as a ‘Jack-and-Master’ (*sensu*Richards *et al.* 2006) allowed rapid range expansion following initial introduction (Atwater *et al.* 2015, 2018; Lakoba and Barney 2020; Lakoba *et al.* 2021*a*), which is limited by strong size/flowering time trade-offs at the range edges. Northward expansion is limited at the intermediate (Mid-Atlantic) range where Johnsongrass is still abundant and common, dropping precipitously to the edge where the climate is too cold for persistence (all rhizomes died in the New York edge garden), and the short summer season elicits strong phenological/fitness trade-offs. At this range edge, even the ‘home’ edge population performed poorly.

The core (Georgia) environment elicited early flowering, smaller individuals with no evidence of local adaptation (i.e. ‘home’ populations did not outperform ‘away’ populations in home core garden), while the intermediate and edge gardens showed later flowering. Despite the ‘aggressive’ nature of this fast-growing rhizomatous perennial, plant competition is strongly limiting everywhere in the first year (Atwater *et al.* 2018); though we have shown large differences in establishment whether starting from seed or rhizome (Atwater *et al.* 2017). We did not evaluate rhizome production, which may have varied across gardens and would be interesting to evaluate in future studies. However, our results suggest that the effects of competition are the strongest in intermediate and edge environments. In contrast to northward expansion, we could not identify what factor(s) limited its westward expansion; transplants in New Mexico exhibited tremendous growth potential with or without supplemental water following initial establishment.

Intermediate populations were able to achieve high performance (biomass and height), not only in their home gardens but also in most of the other gardens, displaying an ability to maintain high performance across a range of environmental conditions. This suggests that the intermediate populations may be functioning as ‘general purpose’ (Baker 1965) or ‘jack-and-master’ genotypes (Richards *et al.* 2006). Previous work with Johnsongrass found that phenotypic plasticity allowed Johnsongrass to perform well in response to different habitats and climatic variations, enabling its rapid spread in the USA (Atwater *et al.* 2015).

### Adaptation at the range edge

While we found evidence of ‘Jack-and-Master’ plasticity in Johnsongrass, this does not allow any species to expand its range indefinitely (Chuang and Peterson 2016). We found evidence of trait differentiation between edge and other populations. The edge populations consistently grew smaller and shorter than the other three populations across all gardens, including their ‘home’ garden. Edge populations also reached reproductive maturity faster than other populations across nearly all gardens, indicating that the edge Johnsongrass populations have been selected to flower earlier but perhaps at the expense of growth. Populations at the northern edge of their range, where the growing season may be shorter due to either cold (northern edge) or moisture (western edge), often prioritize reproduction over vegetative growth in order to complete their life cycle (Chuang and Peterson 2016). However, if the selection is acting on flowering time such that shorter growing seasons favour earlier flowering time (Lande and Arnold 1983; Colautti and Barrett 2010), as suggested by our results, we would have expected earlier flowering time in the edge populations to confer a fitness advantage over the other populations in their respective gardens located at the edge. But, we observed the opposite, as the edge populations under-performed in their home environments as well as in the other environments.

Life history theory predicts that the covariance between traits, in the absence of sufficient genetic diversity for correlated traits, will cause the selection to act on one trait to the detriment of others, resulting in a trade-off (Lande and Arnold 1983; Blows and Hoffmann 2005). Populations at the edge often experience bottlenecks that lead to reduced genetic diversity and fitness (Sexton *et al.* 2009). We have observed Johnsongrass populations to be rare, small and isolated beyond the intermediate range, likely leading to a lack of genetic variation for combinations of traits, resulting in a trade-off between flowering time and size. Further, limited potential for pollen-mediated gene flow among the rather sparsely distributed plants could have limited genetic diversity. While we did not measure genetic diversity in this study, we did observe clear trade-offs between plant size and flowering time. This trade-off can result in a decrease in plant performance and population growth (Colautti *et al.* 2010), which acts to restrict further range expansion beyond the range edge.

### Performance (growth) across the range

We expected a decrease in growth at the range edge, as is a common assumption in species distribution theory (Sexton *et al.* 2009). However, we found overall Johnsongrass growth varied widely across gardens with no consistent decrease in performance at the range edge despite the fact that range-edge accessions themselves were small-statured. Along the longitudinal gradient, we observed an increase in performance from the centre of the range towards the edge. While this should be interpreted with caution as the reported New Mexico performance was from irrigated plots, though there was no appreciable difference in Johnsongrass performance between irrigated and non-irrigated plants (Supplementary Table S4-4, Supplementary Fig. S1).

In addition to apparent performance trade-offs at the range edge (i.e. edge populations under-performed relative to all ‘away’ populations in their home edge garden), the climates of the edge gardens posed varying limitations to Johnsongrass demography. We showed that Johnsongrass populations from across its range can survive and sexually reproduce in New Mexico and New York, well beyond the range centre. However, at the northern edge, no Johnsongrass populations survived the winter, which agrees with our previous work showing rhizome fragments being particularly sensitive to cold temperatures, much more so than seeds (Lakoba *et al.* 2021*b*). At the western edge, all Johnsongrass populations survived the arid growing season and winter, suggesting we had not yet found the climatic limitations to range expansion. However, given the rarity of extant Johnsongrass in New Mexico, and the fact that all known populations occur where water is available yearlong, more information is needed to understand what limits Johnsongrass populations at its Western range edge. Our previous work has shown that seedling establishment is the critical life history transition for Johnsongrass population demography (Atwater *et al.* 2017; Lakoba and Barney 2020), and in this study, we transplanted several-week-old seedlings and watered them for up to 2 weeks. Thus, we hypothesize that seedling germination and/or establishment may play an important role in arid environments. A targeted study of this life stage would be needed to test this hypothesis.

### Biotic interactions

We found strong evidence that climate interacted with competition to determine performance across the range. Weed competition predictably reduced Johnsongrass performance in all gardens and tended to normalize biomass growth. Competition was most intense in the Virginia and New Mexico gardens where Johnsongrass grew largest without competition and had less impact in Georgia, Texas and New York. Though there existed considerable population variation, our results suggest that competition plays a proportionally stronger role in environments where the climate is more favourable for Johnsongrass, which is consistent with the prediction that biotic interactions become more important in less stressful abiotic conditions (Louthan *et al.* 2015). There are several possible explanations for this pattern. For example, less stressful abiotic conditions may be favourable for local species as well as introduced species, resulting in increased competitive pressure on the introduced species or even competitive exclusion (Louthan *et al.* 2015). Importantly, competition did not alter the rank order of population performance at each common garden. Thus, while it had important ecological effects, competition did not appear to differentiate the fitness of different populations along the range. The effect of climate is less commonly evaluated in concert with biotic interactions (Geber 2008), and we are not aware of any studies looking at both factors at the range edge of an invasive species (Holt 2009). Future studies should incorporate the entire life cycle of Johnsongrass, including seed/rhizome germination and seedling establishment (Atwater *et al.* 2017; Lakoba *et al.* 2021*b*).

## Conclusions

The ability to maintain high performance across a wide range of environments via ‘Jack-and-master’ phenotypes has enabled Johnsongrass to invade and rapidly expand its range across a large, ecologically and environmentally diverse swath of the USA. However, Johnsongrass has been unable to become common in drier climates and higher latitudes. These limits to range expansion may be partially explained by a combination of smaller size, resulting from early flowering in the northern and western edges, and the lack of overwinter survival in the northern edge. Abiotic and biotic limitations do not seem to uniformly impact Johnsongrass range expansion and population performance, but they clearly interact in complex ways across large temperature and precipitation gradients. We show the strong role of plant competition in limiting Johnsongrass growth across its range; however, biotic interactions did not change which populations performed best in each garden. Multi-year demographic studies would help elucidate the life history stages that might be limiting Johnsongrass population growth at both peripheries (Metcalf and Pavard 2007).

Latitudinal and longitudinal range edges present unique limitations to future spread. Both edge populations performed poorly relative to other populations across all gardens, including their home edge gardens, suggesting that existing edge populations are not locally adapted and may in fact be maladapted, though this requires further study. Our study highlights the importance of incorporating range core, intermediate and edges in studies of range expansion in invasive species; such continental-scale studies are often called for but rarely done (e.g. Sexton *et al.* 2009). Including the range, edge can provide important insight into biological invasions as well as species distributions and range limits in general (Moran and Alexander 2014). A clear understanding of the drivers and limitations of invasive species range expansion is urgently needed as global change is projected to shift the ranges of invasive species (Bellard *et al.* 2013) and enable some native species to expand beyond their historic boundaries (Essl *et al.* 2019). These interactions at the range edge where abiotic, biotic and demographic factors are strongest will determine the fate of range expansion (Sexton *et al.* 2009). Range expansion at the edge will also present a suite of complex socio-political challenges as species move into novel regions (Essl *et al.* 2019), suggesting more studies of range-expanding native and invasive species should be prioritized.

## Supporting Information

The following additional information is available in the online version of this article –

**Appendix 1**: Calculation of biomass and height relative to the phytometer.

**Appendix 2**: Additional results tables.

**Table S1**. Pair-wise comparisons for significant effects of the mixed-effects models for the latitudinal gradient.

**Table S2**: Pair-wise comparisons for significant effects of the mixed-effects models for the longitudinal gradient.

**Table S3**: Proportion of plants that survived in each population, treatment, and garden.**Appendix 3**: New Mexico Irrigation Sub-experiment.**Table S4**: Results of mixed-effects models assessing the effects of population, irrigation, treatment, and their interactions on cube-root biomass, height, and flowering time of three Johnsongrass populations and one phytometer (control) population planted in a common garden in New Mexico.**Table S5**: Proportion of plants that survived in each population, treatment, and irrigation regime in the New Mexico garden.**Figure S1**: Effects of population, treatment, and garden on Johnsongrass above-ground biomass in the New Mexico garden under two irrigation regimes: irrigated and not irrigated.**Figure S2**: Effects of population, treatment, and garden on Johnsongrass height in the New Mexico garden under two irrigation regimes: irrigated and not irrigated.**Figure S3**: Effects of population, treatment, and garden on Johnsongrass flowering time in the New Mexico garden under two irrigation regimes: irrigated and not irrigated.**Appendix 4**: Effects of competition on Johnsongrass biomass.**Table S6**: Tests for the effect of population, garden, and their interaction on competitive effect along a latitudinal and a longitudinal gradient.**Figure S4**: Results along the latitudinal (temperature) gradient of Johnsongrass’ North American range.**Figure S5**: Results along the longitudinal (precipitation) gradient of Johnsongrass’ North American range.

plad070_suppl_Supplementary_Material

plad070_suppl_Supplementary_Data_S1

plad070_suppl_Supplementary_Data_S2

## Data Availability

All raw data are being made publicly available, DOI forthcoming. Supporting information is included.

## References

[CIT0001] Alexander JM , Edwards PJ. 2010. Limits to the niche and range margins of alien species. Oikos 119:1377–1386.

[CIT0002] Angert AL. 2009. The niche, limits to species’ distributions, and spatiotemporal variation in demography across the elevation ranges of two monkeyflowers. Proceedings of the National Academy of Sciences of the United States of America 106:19693–19698. 19805178 10.1073/pnas.0901652106 PMC2780943

[CIT0003] Atwater DZ , Sezen UU, Goff V, Kong W, Paterson AH, Barney JN. 2015. Reconstructing changes in the genotype, phenotype, and climatic niche of an introduced species. Ecography 39:894–903.

[CIT0004] Atwater DZ , Kim W, Tekiela DR, Barney JN. 2017. Competition and propagule density affect sexual and clonal propagation of a weed. Invasive Plant Science and Management 10:17–25.

[CIT0005] Atwater DZ , Fletcher RA, Dickinson CC, Paterson AH, Barney JN. 2018. Evidence for fine-scale habitat specialisation in an invasive weed. Journal of Plant Ecology 11:rtw124–rtw199.

[CIT0006] Baker HG 1965 Characteristics and modes of origin of weeds. In: Baker HG, Stebbins HG, eds. The genetics of colonizing species. New York, NY: Academic Press, 147–172

[CIT0007] Bates D , Maechler M, Bolker B, Walker S. 2015. Fitting linear mixed-effects models using lme4. Journal of Statistical Software 67:1–48.

[CIT0008] Bellard C , Thuiller W, Leroy B, Genovesi P, Bakkenes M, Courchamp F. 2013. Will climate change promote future invasions? Global Change Biology 19:3740–3748. 23913552 10.1111/gcb.12344 PMC3880863

[CIT0009] Blanquart F , Kaltz O, Nuismer SL, Gandon S. 2013. A practical guide to measuring local adaptation. Ecology Letters 16:1195–1205. 23848550 10.1111/ele.12150

[CIT0010] Blows MW , Hoffmann AA. 2005. A reassessment of genetic limits to evolutionary change. Ecology 86:1371–1384.

[CIT0011] Bontrager M , Angert AL. 2019. Gene flow improves fitness at a range edge under climate change. Evolution Letters 3:55–68. 30788142 10.1002/evl3.91 PMC6369935

[CIT0012] Bridle JR , Vines TH. 2007. Limits to evolution at range margins: when and why does adaptation fail? Trends in Ecology & Evolution 22:140–147. 17113679 10.1016/j.tree.2006.11.002

[CIT0013] Bullock JM , Edwards RJ, Carey PD, Rose RJ. 2000. Geographical separation of two *Ulex* species at three spatial scales: does competition limit species’ ranges? Ecography 23:257–271.

[CIT0014] Chuang A , Peterson CR. 2016. Expanding population edges: theories, traits, and trade-offs. Global Change Biology 22:494–512. 26426311 10.1111/gcb.13107

[CIT0015] Colautti RI , Barrett SCH. 2010. Natural selection and genetic constraints on flowering phenology in an invasive plant. International Journal of Plant Sciences 171:960–971.

[CIT0016] Colautti RI , Barrett SCH. 2013. Rapid adaptation to climate facilitates range expansion of an invasive plant. Science 342:364–366. 24136968 10.1126/science.1242121

[CIT0017] Colautti RI , Eckert CG, Barrett SCH. 2010. Evolutionary constraints on adaptive evolution during range expansion in an invasive plant. Proceedings Biological Sciences 277:1799–1806. 20164098 10.1098/rspb.2009.2231 PMC2871878

[CIT0018] Csergő AM , Salguero-Gómez R, Broennimann O, Coutts SR, Guisan A, Angert AL, Welk E, Stott I, Enquist BJ, McGill B, et al. 2017. Less favourable climates constrain demographic strategies in plants. Ecology Letters 20:969–980. 28609810 10.1111/ele.12794 PMC5575490

[CIT0019] Essl F , Dullinger S, Genovesi P, Hulme PE, Jeschke JM, Katsanevakis S, Kühn I, Lenzner B, Pauchard A, Pyšek P, et al. 2019. A conceptual framework for range-expanding species that track human-induced environmental change. BioScience 69:908–919.

[CIT0020] Gaston KJ. 1990. Patterns in the geographical ranges of species. Biological Reviews 65:105–129.

[CIT0021] Geber MA. 2008. To the edge: studies of species’ range limits. New Phytologist 178:228–230. 18371003 10.1111/j.1469-8137.2008.02414.x

[CIT0022] Hargreaves AL , Samis KE, Eckert CG. 2014. Are species’ range limits simply niche limits writ large? A review of transplant experiments beyond the range. The American Naturalist 183:157–173. 10.1086/674525 24464192

[CIT0023] Holt RD. 2009. Up against the edge: invasive species as testbeds for basic questions about evolution in heterogeneous environments. Molecular Ecology 18:4347–4348. 19845861 10.1111/j.1365-294X.2009.04358.x

[CIT0024] Holt RD , Keitt TH, Lewis MA, Maurer BA, Taper ML. 2005. Theoretical models of species’ borders: single species approaches. Oikos 108:18–27.

[CIT0025] Jump AS , Woodward FI. 2003. Seed production and population density decline approaching the range-edge of *Cirsium* species. The New Phytologist 160:349–358. 33832171 10.1046/j.1469-8137.2003.00873.x

[CIT0026] Kirkpatrick M , Barton NH. 1997. Evolution of a species’ range. The American Naturalist 150:1–23. 10.1086/286054 18811273

[CIT0027] Körner C , Basler D, Hoch G, Kollas C, Lenz A, Randin CF, Vitasse Y, Zimmermann NE. 2016. Where, why and how? Explaining the low-temperature range limits of temperate tree species. Journal of Ecology 104:1076–1088.

[CIT0028] Lakoba VT , Barney JN. 2020. Home climate and habitat drive ecotypic stress response differences in an invasive grass. AoB Plants 12:plaa062. 33408848 10.1093/aobpla/plaa062 PMC7770431

[CIT0029] Lakoba VT , Atwater DZ, Thomas VE, Strahm BD, Barney JN. 2021a. A global invader’s niche dynamics with intercontinental introduction, novel habitats, and climate change. Global Ecology and Conservation 31:e01848.

[CIT0030] Lakoba VT , Welbaum GE, Seiler JR, Barney JN. 2021b. A perennial invader’s seed and rhizome differ in cold tolerance and apparent local adaptation. NeoBiota 70:1–21.

[CIT0031] Lande R , Arnold SJ. 1983. The measurement of selection on correlated characters. Evolution 37:1210–1226. 28556011 10.1111/j.1558-5646.1983.tb00236.x

[CIT0032] Lenth R 2019 Emmeans: estimated marginal means, aka least-squares means. R package version 1.3.4.

[CIT0033] Louthan AM , Doak DF, Angert AL. 2015. Where and when do species interactions set range limits? Trends in Ecology & Evolution 30:780–792. 26525430 10.1016/j.tree.2015.09.011

[CIT0034] Louthan AM , Pringle RM, Goheen JR, Palmer TM, Morris WF, Doak DF. 2017. Aridity weakens population-level effects of multiple species interactions on *Hibiscus meyeri*. Proceedings of the National Academy of Sciences 115:543–548. 10.1073/pnas.1708436115 PMC5776961 29284748

[CIT0035] Maity A , Young B, Subramanian N, Bagavathiannan M. 2022. Pollen-mediated transfer of herbicide resistance between johnsongrass (*Sorghum halepense*) biotypes. Scientific Reports 12:7663. 35538136 10.1038/s41598-022-11713-8 PMC9091218

[CIT0036] McDonald A , Riha S, DiTommaso A, DeGaetano A. 2009. Climate change and the geography of weed damage: analysis of US maize systems suggests the potential for significant range transformations. Agriculture, Ecosystems & Environment 130:131–140.

[CIT0037] McWhorter CG. 1961. Morphology and development of Johnsongrass plants from seeds and rhizomes. Weeds 9:558–562.

[CIT0038] Metcalf CJE , Pavard S. 2007. Why evolutionary biologists should be demographers. Trends in Ecology and Evolution 22:205–212. 17174004 10.1016/j.tree.2006.12.001

[CIT0039] Moran EV , Alexander JM. 2014. Evolutionary responses to global change: lessons from invasive species. Ecology Letters 17:637–649. 24612028 10.1111/ele.12262

[CIT0040] Paterson AH , Kong WQ, Johnston RM, Nabukalu P, Wu G, Poehlman WL, Goff VH, Isaacs K, Lee T-H, Guo H, et al. 2020. The evolution of an invasive plant, *Sorghum halepense* L (‘Johnsongrass’). Frontiers in Genetics 11:317. 32477397 10.3389/fgene.2020.00317 PMC7240026

[CIT0041] Pironon S , Papuga G, Villellas J, Angert AL, García MB, Thompson JD. 2017. Geographic variation in genetic and demographic performance: new insights from an old biogeographical paradigm. Biological Reviews of the Cambridge Philosophical Society 92:1877–1909. 27891813 10.1111/brv.12313

[CIT0042] R Core Team 2018 R: a language and environment for statistical computing. Vienna: R Foundation for Statistical Computing, https://www.R-project.org/

[CIT0043] Richards CL , Bossdorf O, Muth NZ, Gurevitch J, Pigliucci M. 2006. Jack of all trades, master of some? On the role of phenotypic plasticity in plant invasions. Ecology Letters 9:981–993. 16913942 10.1111/j.1461-0248.2006.00950.x

[CIT0044] Rout ME , Chrzanowski TH, Smith WK, Gough L. 2013. Ecological impacts of the invasive grass *Sorghum halepense* on native tallgrass prairie. Biological Invasions 15:327–339.

[CIT0045] Sagarin RD , Gaines SD. 2002. The ‘abundant centre’ distribution: to what extent is it a biogeographical rule? Ecology Letters 5:137–147.

[CIT0046] Sanford E , Roth MS, Johns GC, Wares JP, Somero GN. 2003. Local selection and latitudinal variation in a marine predator-prey interaction. Science (New York, N.Y.) 300:1135–1137. 12750518 10.1126/science.1083437

[CIT0047] Sexton JP , McInyre PJ, Angert AL, Rice KJ. 2009. Evolution and ecology of species range limits. Annual Review of Ecology, Evolution, and Systematics 40:415–436.

[CIT0048] Sezen UU , Barney JN, Atwater DZ, Pederson GA, Pederson JF, Chandler JM, Cox TS, Cox S, Dotray P, Kopec D, et al. 2016. Multi-phase US spread and habitat switching of a post-columbian invasive, *Sorghum halepense*. PLoS One 11:e0164584–e0164514. 27755565 10.1371/journal.pone.0164584 PMC5068735

[CIT0049] Smith AL , Atwater DZ, Kim W, Haak DC, Barney JN. 2021. Invasive plant rhizome production and competitiveness vary based on neighbor identity. Journal of Plant Ecology 14:638–647.

[CIT0050] Stanton-Geddes J , Tiffin P, Shaw RG. 2012. Role of climate and competitors in limiting fitness across range edges of an annual plant. Ecology 93:1604–1613. 22919907 10.1890/11-1701.1

[CIT0051] Strobl K , Schmidt C, Kollmann J. 2018. Selecting plant species and traits for phytometer experiments. The case of peatland restoration. Ecological Indicators 88:263–273.

[CIT0052] Theoharides KA , Dukes JS. 2007. Plant invasion across space and time: factors affecting nonindigenous species success during four stage of invasion. The New Phytologist 176:256–273. 17822399 10.1111/j.1469-8137.2007.02207.x

[CIT0053] Thomas CD. 2010. Climate, climate change and range boundaries. Diversity and Distributions 16:488–495.

[CIT0054] Warwick SI , Black LD. 1983. The biology of Canadian weeds. 61. *Sorghum halepense* (L) Pers. Canadian Journal of Plant Science 63:997–1014.

[CIT0055] Warwick SI , Thompson BK, Black LD. 1984. Population variation in *Sorghum halepense*, Johnson grass, at the northern limits of its range. Canadian Journal of Botany 62:1781–1790.

[CIT0056] Warwick SI , Phillips D, Andrews C. 1986. Rhizome depth: the critical factor in winter survival of *Sorghum halepense* (L) Pers (Johnson grass). Weed Research 26:381–388.

[CIT0057] Watling JI , Orrock JL. 2010. Measuring edge contrast using biotic criteria helps define edge effects on the density of an invasive plant. Landscape Ecology 25:69–78.

[CIT0058] Younginger BS , Sirová D, Cruzan MB, Ballhorn DJ. 2017. Is biomass a reliable estimate of plant fitness? Applications in Plant Sciences 5:1600094. 10.3732/apps.1600094 PMC5315378 28224055

