# Supplementary material for: Adaptive constraints at the range edge of a widespread and expanding invasive plant
Source: AoB Plants. 2023 Nov 5;15(6):plad070. doi: 10.1093/aobpla/plad070 (PMC10651072; doi:10.1093/aobpla/plad070)
Supplement: plad070_suppl_Supplementary_Material [file plad070_suppl_supplementary_material.pdf]

Additional Methods and Results

Appendix 1: Calculation of Biomass and Height Relative to the Phytometer

We calculated the above-ground biomass and height relative to the phytometer of each population in each block and treatment using the following equations:

ln(biomass relative to PHYT) = $ln\left(\frac{Biomass_{p,b,t}}{Biomass_{PHYT,b,t}}\right)$

ln(height relative to PHYT) = $ln\left(\frac{Height_{p,b,t}}{Height_{PHYT,b,t}}\right)$

Where p is population (GA, VA, TX, NY, or NM), b is block (1–50), t is treatment (bare-ground or weed-competition), and $PHYT$ is the phytometer ("control" population).

If a phytometer plant was missing from one of the blocks, we used the mean value of above-ground biomass and height of phytometer plants in the same garden and the same treatment.

Appendix 2: Additional Results Tables

Table S1: Pair-wise comparisons for significant effects of the mixed-effects models for the latitudinal gradient. Each model included the fixed effects: population, garden, and treatment and their interactions. Pair-wise comparisons were conducted within the levels of the interacting predictors. Lower case letters in brackets indicate pair-wise differences using the Tukey method for multiple comparisons. Levels within a factor are always ordered from smallest value to largest value.

Explanatory Variable	Significant Fixed-effect	Contrasts
Cube-root Biomass	Pop. × Gard.	In Georgia: NM(a), NY(ab), VA(ab), TX(ab), GA(ab), PHYT(b)
		In Virginia: NM(a), NY(a), GA(ab), TX(bc), VA(c), PHYT(d)
		In New York: NM(a), NY(a), VA(a), TX(a), GA(a), PHYT(b)
	Treat. × Gard.	In Georgia: Weeds(a), Bare-ground(b)
		In Virginia: Weeds(a), Bare-ground(b)
		In New York: Weeds(a), Bare-ground(b)
Height	Population	NY(a), NM(ab), GA(bc), TX(cd), VA(de), PHYT(e)
	Treat. × Gard.	In Georgia: Weeds(a), Bare-ground(b)
		In Virginia: Weeds(a), Bare-ground(b)
		In New York: Weeds(a), Bare-ground(b)
ln(Biomass Relative to PHYT)	Population	NM(a), NY(ab), VA(ab), TX(ab), GA(b)
ln(Height Relative to PHYT)	Population	NY(a), NM(ab), GA(bc), TX(cd), VA(d)

Flowering Time	Population	NY(a), PHYT(a), NM(ab), TX(bc), GA(c), VA(c)
	Garden	Georgia(a), New York(b), Virginia(b)

Table S2: Pair-wise comparisons for significant effects of the mixed-effects models for the longitudinal gradient. Each model included the fixed effects: population, garden, and treatment and their interactions. Pair-wise comparisons were conducted within the levels of the interacting predictors. Lower case letters in brackets indicate pair-wise differences using the Tukey method for multiple comparisons. Levels within a factor are always ordered from smallest value to largest value.

Explanitory Variable	Significant Fixed-effect	Contrasts
Cube-root Biomass	Pop. × Gard.	In Georgia: NM(a), NY(a), VA(a), TX(a), GA(a), PHYT(a)
		In Texas: NY(a), NM(ab), VA(bc), GA(bc), TX(cd), PHYT(d)
		In New Mexico: NY(a), NM(a), TX(a), GA(ab), VA(bc), PHYT(c)
	Treat. × Gard.	In Georgia: Weeds(a), Bare-ground(b)
		In Texas: Weeds(a), Bare-ground(b)
		In New Mexico: Weeds(a), Bare-ground(b)
Height	Pop. × Gard.	In Georgia: NY(a), NM(a), TX(a), GA(a), VA(b), PHYT(b)
		In Texas: NY(a), NM(ab), GA(ab), VA(bc), PHYT(bc), TX(c)
		In New Mexico: NY(a), NM(a), GA(a), TX(ab), PHYT(b), VA(b)
	Treat. × Gard.	In Georgia: Weeds(a), Bare-ground(b)
		In Texas: Weeds(a), Bare-ground(b)
		In New Mexico: Weeds(a), Bare-ground(b)
ln(Biomass Relative to PHYT)	Population	NY(a), NM(ab), TX(bc), VA(c), GA(c)
	Treat. × Gard.	In Georgia: Weeds(a), Bare-ground(b)
		In Texas: Weeds(a), Bare-ground(a)
		In New Mexico: Weeds(a), Bare-ground(b)

ln(Height Relative to PHYT)	Population	NY(a), NM(ab), GA(bc), TX(cd), VA(d)
	Treat. × Gard.	In Georgia: Weeds(a), Bare-ground(a)
		In Texas: Bare-ground(a), Weeds(b)
		In New Mexico: Weeds(a), Bare-ground(a)
Flowering Time	Pop. × Gard.	In Georgia: NY(a), PHYT(ab), NM(abc), TX(abc), VA(bc), GA(c)
		In Texas: NY(a), NM(a), GA(a), VA(a), PHYT(a), TX(a)
		In New Mexico: NM(a), PHYT(a), NY(ab), TX(bc), GA(c), VA(c)
	Treatment	Bare-ground(a), Weeds(b)

Table S3: Proportion of plants that survived in each population, treatment, and garden. Survival was recorded at the end of the growing season in 2017 and again in Spring 2018.

	Georgia		New Mexico		New York		Texas		Virginia	
	End of Season Survival	Over-winter Survival	End of Season Survival	Over-winter Survival	End of Season Survival	Over-winter Survival	End of Season Survival	Over-winter Survival	End of Season Survival	Over-winter Survival
PHYT										
Bare-Ground	1.00	0.40	1.00	1.00	1.00	0	1.00	1.00	1.00	0.33
Weeds	0.89	0.63	1.00	0.90	1.00	0	1.00	1.00	1.00	0.10
GA										
Bare-Ground	1.00	0.60	1.00	1.00	1.00	0	1.00	1.00	0.80	1.00
Weeds	0.67	0.17	1.00	1.00	0.89	0	1.00	1.00	0.90	0.89
NM										
Bare-Ground	0.90	0.89	1.00	1.00	0.90	0	1.00	1.00	1.00	1.00
Weeds	0.38	0.67	1.00	1.00	0.90	0	1.00	1.00	1.00	0.70
NY										
Bare-Ground	1.00	0.90	1.00	1.00	1.00	0	1.00	1.00	1.00	1.00
Weeds	0.56	0.80	1.00	1.00	0.70	0	1.00	1.00	1.00	0.80
TX										
Bare-Ground	1.00	0.80	1.00	1.00	0.75	0	1.00	1.00	0.90	1.00
Weeds	0.67	0.67	0.85	1.00	0.86	0	1.00	1.00	1.00	1.00
VA										
Bare-Ground	0.90	0.89	1.00	1.00	0.89	0	1.00	1.00	0.88	1.00
Weeds	0.67	0.83	1.00	1.00	1.00	0	1.00	1.00	1.00	0.88
Overall										
Bare-Ground	0.97	0.74	1.00	1.00	0.93	0	1.00	1.00	0.93	0.89
Weeds	0.64	0.62	0.98	0.98	0.88	0	1.00	1.00	0.98	0.72

Appendix 3: New Mexico Irrigation Sub-experiment

In addition to the 10 main blocks in New Mexico, which were irrigated with supplemental water throughout the duration of the experiment, we included five additional blocks with the same experimental set up, except that they received no irrigation beyond the initial two-week establishment period. Because seedlings of some populations were in short supply, only populations that had additional seedlings left over from the main 10-block experiment were planted into the five additional, non-irrigated blocks. No seedlings from the VA and TX populations were left; thus, only the phytometer and populations from NY, GA, and NM were included in the non-irrigated blocks.

We used mixed-effects linear models to assess the fixed effects of treatment (bare-ground and weed-competition), population, and irrigation, and their interactions, on above-ground biomass, height, and flowering time. The random effect of block was included in all models.

There were significant effects of population, irrigation, and treatment on biomass and height (Table S4). The NY and NM populations grew the smallest and the phytometer grew the largest, and all populations grew equally shorter than the phytometer (Figs. S1 and S2). Weed-competition decreased biomass compared to bare-ground, and plants growing in the irrigated blocks had greater biomass compared to those in the non-irrigated blocks (Fig. S1). For height, the effect of treatment depended on irrigation (Table S4). Weed-competition decreased height in both irrigated and non-irrigated blocks, but the magnitude by which weed-competition decreased height in the non-irrigated blocks was greater than in the irrigated blocks (Fig. S2). This suggests a possible compounding effect of weed competition on height outside of irrigated areas in New Mexico. For flowering time, plants in the irrigated blocks flowered earlier than in the non-irrigated blocks, and the effect of population depended on treatment (Table S4, Fig. S3).

All plants in the non-irrigated blocks survived until the end of the season and most survived over the winter to emerge the following spring. Over-winter mortality only occurred in the NY population and the phytometer in the weed-competition treatment (Table S5). Also, 100% of plants flowered by the end of the season in the non-irrigated blocks.

Even though a lack of irrigation reduced both biomass and height and delayed flowering time, the results from this sub-experiment suggest that, as long as Johnsongrass seedlings are able to establish in non-irrigated areas in New Mexico, they are not only likely to survive and reproduce within one growing season, but they can persist to the following year.

Table S4: Results of mixed-effects models assessing the effects of population, irrigation, treatment, and their interactions on cube-root biomass, height, and flowering time of three Johnsongrass populations and one phytometer (control) population planted in a common garden in New Mexico. All models included the random effect of block. The *F*-statistic was calculated using Type III sum of squares.

	SS	*df*	*F*	*P*
Cube-root Biomass				
**Population**	**176.09**	**3**	**17.25**	**<0.001**
**Irrigation**	**21.56**	**1**	**6.34**	**0.024**
**Treatment**	**828.94**	**1**	**243.59**	**<0.001**
Pop. × Irrig.	4.23	3	0.41	0.743
Pop. × Treat.	6.88	3	0.67	0.571
Irrig. × Treat.	1.89	1	0.56	0.458
Pop. × Irrig. × Treat.	22.79	3	2.23	0.090
Height				
**Population**	**12435**	**3**	**7.68**	**<0.001**
**Irrigation**	**24297**	**1**	**45.00**	**<0.001**
**Treatment**	**17607**	**1**	**32.61**	**<0.001**
Pop. × Irrig.	2222	3	1.37	0.256
Pop. × Treat.	1100	3	0.68	0.567
**Irrig. × Treat.**	**3716**	**1**	**6.88**	**0.010**
Pop. × Irrig. × Treat.	3691	3	2.28	0.084
Flowering Time				
**Population**	**2448.83**	**3**	**13.34**	**<0.001**
**Irrigation**	**429.39**	**1**	**7.02**	**0.009**
Treatment	0.37	1	0.01	0.939
Pop. × Irrig.	376.65	3	2.05	0.112
**Pop. × Treat.**	**633.03**	**3**	**3.45**	**0.020**
Irrig. × Treat.	80.26	1	1.31	0.255
Pop. × Irrig. × Treat.	189.11	3	1.03	0.383

Table S5: Proportion of plants that survived in each population, treatment, and irrigation regime in the New Mexico garden. Survival was recorded at the end of the growing season in 2017 and again in Spring 2018.

	End of Season Survival		Winter Survival	
	Irrigated	Non-irrigated	Irrigated	Non-irrigated
PHYT				
Bare-Ground	1.00	1.00	1.00	1.00
Weeds	1.00	1.00	0.90	0.67
GA				
Bare-Ground	1.00	1.00	1.00	1.00
Weeds	1.00	1.00	1.00	1.00
NM				
Bare-Ground	1.00	1.00	1.00	1.00
Weeds	1.00	1.00	1.00	1.00
NY				
Bare-Ground	1.00	1.00	1.00	1.00
Weeds	1.00	1.00	1.00	0.75
TX				
Bare-Ground	1.00	---	1.00	---
Weeds	0.86	---	1.00	---
VA				
Bare-Ground	1.00	---	1.00	---
Weeds	1.00	---	1.00	---

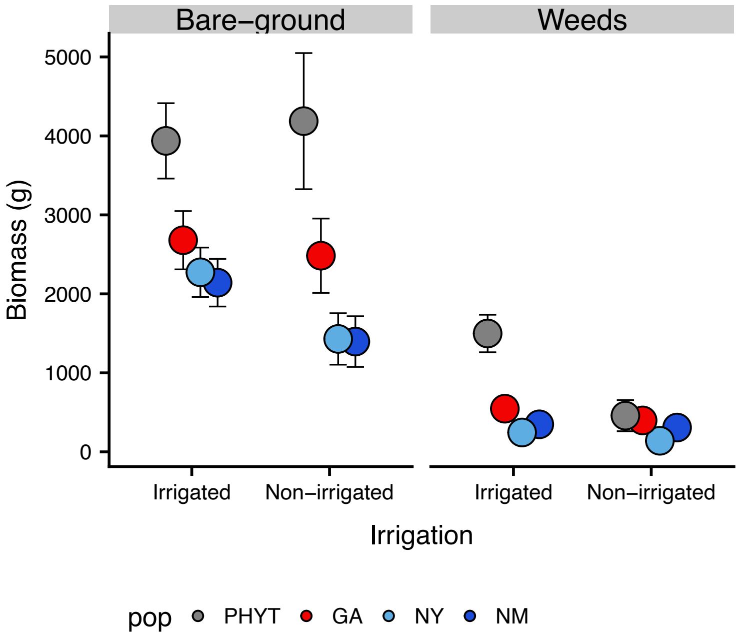

Figure S1: Effects of population, treatment, and garden on Johnsongrass above-ground biomass in the New Mexico garden under two irrigation regimes: irrigated and not irrigated. Points are estimated marginal means and error bars show standard error. The Georgia population is represented by red, the New York population by light blue, the New Mexico population by darker blue, and the phytometer (control population) by gray.

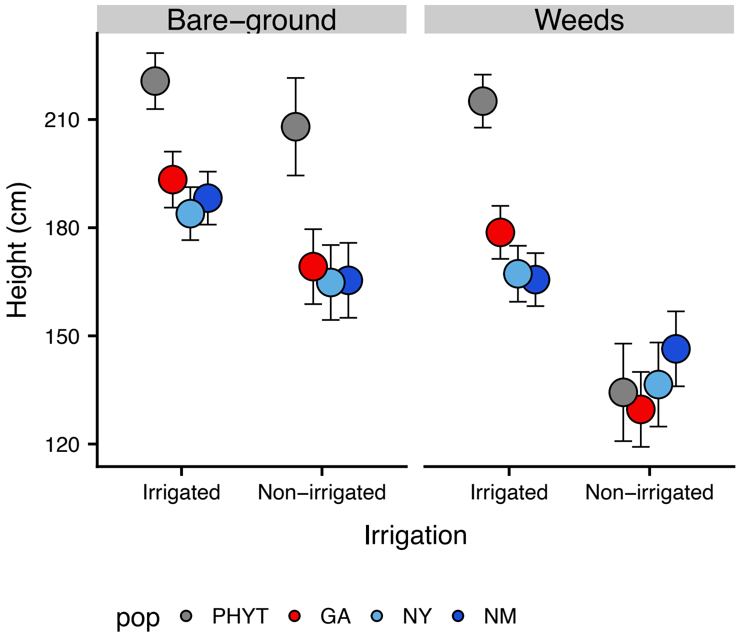

Figure S2: Effects of population, treatment, and garden on Johnsongrass height in the New Mexico garden under two irrigation regimes: irrigated and not irrigated. Points are estimated marginal means and error bars show standard error. The Georgia population is represented by red, the New York population by light blue, the New Mexico population by darker blue, and the phytometer (control population) by gray.

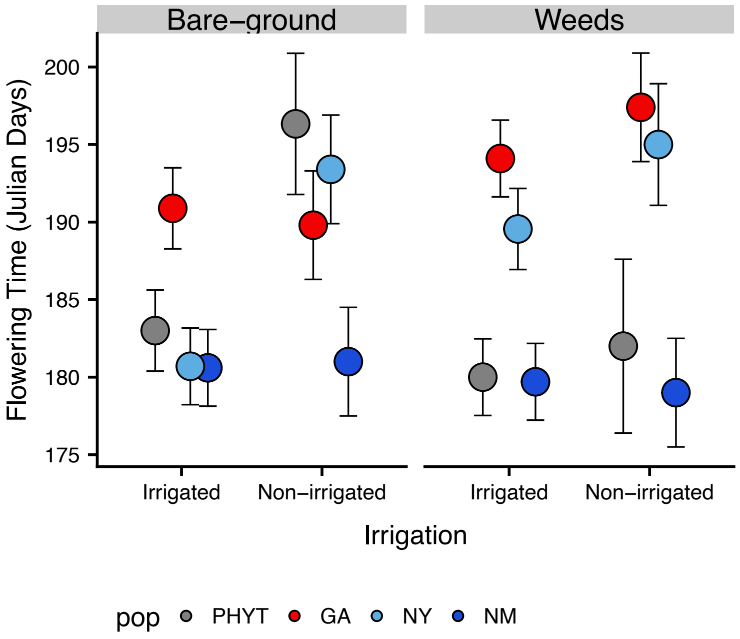

Figure S3: Effects of population, treatment, and garden on Johnsongrass flowering time in the New Mexico garden under two irrigation regimes: irrigated and not irrigated. Points are estimated marginal means and error bars show standard error. The Georgia population is represented by red, the New York population by light blue, the New Mexico population by darker blue, and the phytometer (control population) by gray.

Appendix 4: Effects of competition on Johnsongrass biomass

To investigate a potential interaction between biotic effects (competition) and climate, we calculated the logarithm of the response ratio (lnRR), which is the ratio of performance between plants grown in competition with weeds and plants grown in the absence of weed competition (citation). We calculated the lnRR for biomass using the equation:

$$lnRR = \ln\left(\frac{Biomass_{Comp}}{Biomass_{Bare}}\right)$$

Where $Biomass_{Comp}$ is the biomass of individuals in the weed competition treatment and $Biomass_{Bare}$ is the biomass of individuals in the bare-ground treatment. We calculated lnRR for each population within each block. The lnRR can be interpreted as the intensity of the effect of weed competition on Johnsongrass. A lnRR < 0 indicates a competitive effect and lnRR > 0 indicates a facilitative effect of the resident plant community on Johnsongrass performance. As lnRR deviates from 0, it is indicative of increasing intensity of the effect of weed competition. We used general, mixed-effects models to investigate the fixed effects of population, garden, and their interaction on the lnRR of above-ground biomass. We also included the random effect of block in the model.

Table S6: Tests for the effect of population, garden, and their interaction on competitive effect along a latitudinal and a longitudinal gradient. *F*-statistic for the general linear models was calculated using Type III sum of squares.

	**Latitudinal Gradient**		
	Competitive Effect		
	SS	*F*	*P*
Pop.	7.42	1.65	0.169
Garden	34.54	15.37	< **0.001**
Pop. x Gard.	4.44	0.493	0.858

	**Longitudinal Gradient**		
	Competitive Effect		
	SS	*F*	*P*
Pop.	6.35	1.92	0.115
Garden	128.61	77.67	< **0.001**
Pop. x Gard.	9.79	1.48	0.178

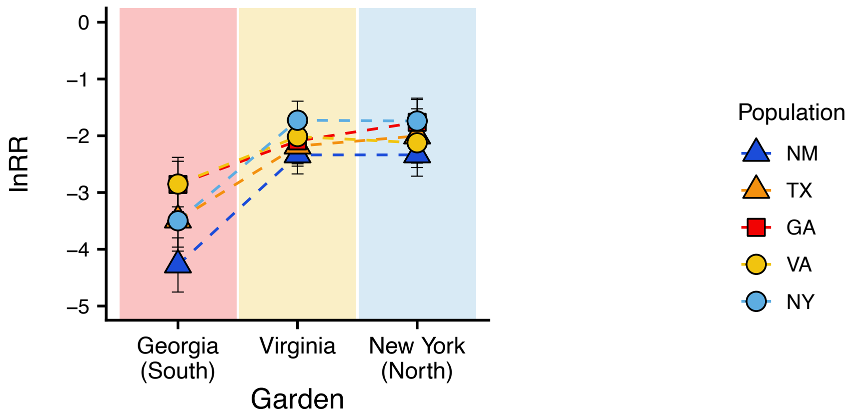

Figure S4: Results along the latitudinal (temperature) gradient of Johnsongrass' North American range. The effect of population and garden on the impact of weed competition on Johnsongrass biomass. Points are estimated marginal means and error bars show standard error. The red square represents the Georgia population. Circles represent populations originating along the latitudinal gradient–Virginia (yellow) and New York (light blue). Triangles represent populations originating along the longitudinal gradient–Texas (orange) and New Mexico (darker blue).

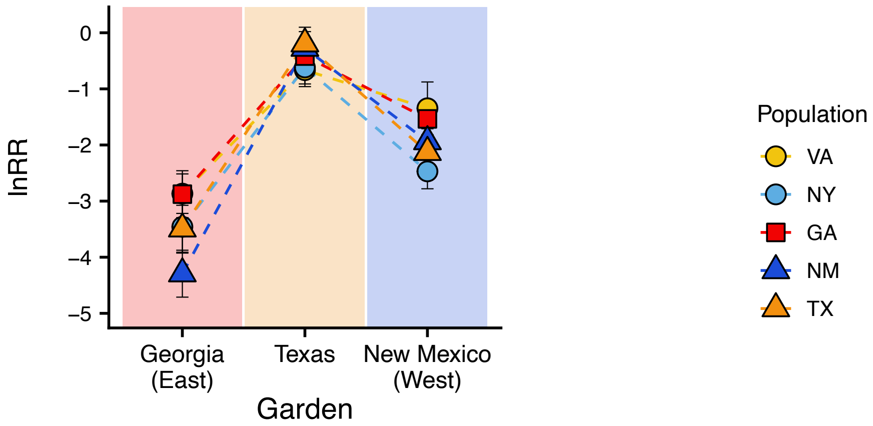

Figure S5: Results along the longitudinal (precipitation) gradient of Johnsongrass' North American range. The effect of population and garden on the impact of weed competition on Johnsongrass biomass. Points are estimated marginal means and error bars show standard error. Circles represent populations originating along the latitudinal gradient–Virginia (yellow) and New York (light blue). Triangles represent populations originating along the longitudinal gradient–Texas (orange) and New Mexico (darker blue).
